# Hydration of Ordinary Portland Cement in Presence of Lead Sorbed on Ceramic Sorbent

**DOI:** 10.3390/ma12010019

**Published:** 2018-12-21

**Authors:** Martin Keppert, Lenka Scheinherrová, Miloš Jerman, Barbora Doušová, Libor Kobera, Jiří Brus, Robert Černý

**Affiliations:** 1 Department of Materials Engineering and Chemistry, Faculty of Civil Engineering, Czech Technical University in Prague, Thákurova 7, 166 29 Praha 6, Czech Republic; lenka.scheinherrova@fsv.cvut.cz (L.S.); milos.jerman@fsv.cvut.cz (M.J.); cernyr@fsv.cvut.cz (R.Č.); 2 Department of Solid State Chemistry, Faculty of Chemical Technology, University of Chemistry and Technology Prague, Technická 5, 166 28 Praha 6, Czech Republic; barbora.dousova@vscht.cz; 3 Department of NMR Spectroscopy, Institute of Macromolecular Chemistry of Czech Academy of Sciences, v.v.i., Heyrovského nám. 2, 162 06 Praha 6, Czech Republic; kobera@imc.cas.cz (L.K.); brus@imc.cas.cz (J.B.)

**Keywords:** lead removal, sorption, hydration, calorimetry, thermogravimetry, MAS NMR spectroscopy

## Abstract

Lead, a highly toxic element, is frequently present in various solid wastes as well as in industrial effluents. Sorption with a low cost sorbent is a simple way of Pb removal from liquid streams, but stabilization of spent sorbent has to be ensured in order to prevent Pb leaching out and possible environmental contamination. In previous research, ceramic sorbent, generated as waste product in brick industry, was tested as sorbent and proved high sorption capacity for lead. Lead was sorbed partially as hydrocerussite and partially as adsorbed surface layer. The Pb leaching from sorbent was very high and thus further immobilization of sorbent was necessary. Lead, as well as other heavy metals, is known as retarder of the hydration process of Ordinary Portland Cement (OPC), used for the immobilization. In this paper, influence of sorbed Pb and PbO, as reference compound, on cement hydration was studied by calorimetry, thermogravimetry and Magic Angle Spinning Nuclear Magnetic Resonance Spectroscopy (MAS NMR). The sorbed lead was found to be less detrimental to hydration retardation due to the lower solubility of precipitated hydrocerussite in basic environment compared to PbO, which forms plumbate anion.

## 1. Introduction

Lead belongs, due to its high toxicity, to elements which environmental emissions and occurrence are carefully monitored. Lead exposure is responsible for effects on nervous system, the reproductive system and kidneys. Lead gets collected in tissues; especially children are vulnerable to lead exposure, since lead is more easily absorbed into growing bodies. Children’s lead intoxication is also linked to hyperactivity and mental retardation [1,2]. For that reasons, strict limits for lead content in drinking water are applied; in EU countries the maximum lead content in drinking water is 10 μg/L, in United States MCL system 6 μg/L is required [3]. Sources of Pb environmental contamination were reviewed in [4]; lead is present in industrial waste waters, e.g., from acid–lead battery production and processing [5] (22 mg/L) or metallurgy [6]. However, due to relatively high volatility of Pb compounds, lead is entering ecosystems also through atmospheric precipitation [7]. An important source of lead in drinking water is also corrosion of lead pipes in older buildings [8]; another intensive source of lead (and possibly other heavy metals) in hydrosphere can be acid mine drainage from sulfidic ore mines (900 μg/L) [9]. The industrial effluent limit for Pb in EU countries is 500 μg/L [10].

There are numerous methods, which can be used for heavy metals (HM) removal from waste waters in order to meet demand requirements; widely used are precipitation and filtration of an insoluble compound of HM (e.g., hydroxide), membrane or electrochemical separation methods [2,4]. Sorption—i.e., entrapment of a contaminant on surface of a solid sorbent—represents a technologically and economically feasible solution, especially when a low cost sorbent is used [11]. Conventionally, activated carbon, zeolites, and clay minerals have been widely used as sorbents thanks to their high specific surface area and suitable chemical composition for adsorption of organic (activated carbon) and inorganic pollutants from both liquid and gaseous streams [12]. Concerning the lead sorption from liquid effluents, Uddin [13] reviewed range of papers dealing with sorption of HM on clay minerals; the found sorption capacities have ranged from ca. 1 to 238 mg/g, reflecting the variability of natural and modified clay minerals. Similarly to clays, zeolites are also characterized by high specific surface area and cation exchange capacity, determining its high sorption capacity for HM [14]. The Pb sorption capacity on activated carbon from palm oil mill effluent was found to be 94 mg/g [15]. Currently, activated carbon is frequently employed for sorption; such banana peels, with a Pb sorption capacity 247 mg/g [16]. Recently, sorption of pollutants on sorbents of microbial and plant origin [17], as well as on nanomaterials [18] are widely studied. Highly favorable, from the environmental point of view, is application of wastes as sorbents for heavy metals. Grace et al. [19] reviewed papers on HM sorption on blast furnace slag, mud from Bayer process and fly ash. High potential, due to their high amount, has sorption on construction and demolition waste [20] or recycled glass [21].

General requirements of a sorbent are high sorption capacity for the desired contaminant, reasonable price, and possibility to recycle the spent sorbent or to stabilize it in order to prevent leaching of contaminant out from the stored sorbent. The present paper deals with sorption of lead on waste red-clay ceramic powder; its sorption ability for Pb, as well as for other elements, has been tested [22]. The ceramic powder is alongside a pozzolana, i.e., it is able to react with Ca(OH)_2_ to C-S-H and C-A-H hydration products and thus be used as active component in blended cements [23]. Even though there are numerous methods for immobilization of toxic materials to prevent leaching of hazardous species [24], the immobilization by help of cement is usually the first choice, especially when the sorbent is pozzolanic material. Unfortunately, heavy metals, including lead, are known to be retarders of cement hydration process [25]; the following mechanism was proposed: hydration retardation is caused by conversion of a HM hydroxide to hydroxyl compound, e.g., Pb(OH)_2_ to plumbate anion PbO_2_^2−^, further precipitating in Ca^2+^ and OH^−^ rich environment as CaPb_2_(OH)_6_·*y*H_2_O. This precipitation reduces Ca^2+^ and OH^−^ concentration in solution and thus delays the reaching of ca. 1.4 supersaturation needed for portlandite (Ca(OH)_2_) and C-S-H gel precipitation [26,27]. Another explanation of retardation lies in formation of an insoluble HM precipitate (e.g., Pb(OH)_2_) on the surface of cement grains [28].

Barbir et al. [29] conducted calorimetry measurements in system CEM I + PbO and found that hydration delay is proportional to the PbO dose. Effect of various Pb compounds on hydration delay was compared by means of NMR relaxometry [30]; PbO was found to have the worse effect, while Pb_3_O_4_ and especially PbO_2_ were neutral. The lead retarding effect can be overcome by help of chloride ion, but with negative impact on the total hydration extent [31]. When several heavy metals were added in soluble form (nitrate) to cement paste, Cu and Pb caused refinement of pore system (while Cd, Ni, and Zn did not), which was explained by their absorption in C-S-H gel [32], which has a positive impact on the stabilization of Pb in the cementitious matrix [33]. Heavy metals are also frequently stabilized/solidified in a geopolymer matrix; Pb added in soluble form to fly ash geopolymer synthesis mixture caused depolymerization of aluminosilicate gel and consequent decrease of strength, but still the immobilization was successful [34]. Koplík et al. [35] observed formation of Pb(OH)_2_ coating on edges of grains in fly ash/blast furnace slag geopolymer. Magnesium-potassium-phosphate cement (MKPC) was tested for Pb stabilization; precipitation of insoluble lead phosphate and chloro-phosphate took place in this environment and any hardening delay was not observed, compared to conventional OPC stabilization matrix [36].

The goal of the study is to describe the influence of lead sorption on ceramic sorbent on hydration course of Ordinary Portland Cement and to compare it with effect of the corresponding amount of PbO. The significance of the paper lies in thorough description of lead speciation on the ceramic sorbent and in description of influence of the sorbed Pb on OPC hydration.

## 2. Materials and Methods

### 2.1. Experimental Program and Materials

The performed experimental program consisted in study of hydration process in cementitious mixtures summarized in Table 1. The control mixture contained just Ordinary Portland Cement (CEM I 42.5 R from Mokrá cement plant, Mokrá, Czech Republic) and water (w/c 0.48). Lead was added in two forms; either as sorbed on ceramic sorbent—samples CEM + PbSorb-*x*, where *x* stands for mass fraction of Pb per OPC (*x* = 0.5 and 2), or as PbO (CEM + Pb-*x*). PbO was used as an insoluble Pb compound without any specific anion, which could also affect the hydration course. In order to evaluate hydration behavior of the ceramic sorbent itself, mixtures without sorbed Pb were prepared (CEM + Sorb-*x*). The pastes were casted to cubic (20 mm) molds and cured in 100% relative humidity and laboratory temperature.

Chemical composition (examined by X-ray fluorescence (XRF) spectroscopy) of the used raw materials is presented in Table 2. The sorbent was generated originally as waste brick dust, collected in production of perforated brick blocks (brick factory Heluz Libochovice, Libochovice, Czech Republic). The *d*_50_ of the sorbent is 6.9 μm and its specific surface area (by BET method) is 4.8 m^2^/g. The sorbent with sorbed lead (PbSorb) contained 13% (by mass) of lead (expressed as PbO). The sorption of lead on ceramic sorbent was performed by batch experiment with 0.5 M/L solution of Pb(NO_3_)_2_; the sorbed amount of Pb represents its maximum content on the given sorbent. More details of sorption experiment were published elsewhere [37]; the Pb sorption data obeyed Langmuir isotherm. The leaching of Pb from the sorbent (PbSorb), as well as from the cementitious pastes, was tested by means of 24 h shaking in water (*L*/*S* = 10) and Atomic Absorption Spectrometry (Agilent 280 FS, Agilent, Santa Clara, CA, USA) analysis of Pb content in the leachate.

### 2.2. Experimental Methods

The chemical composition of used materials was examined by XRF spectroscopy (Thermo ARL 9400 XP, (ThermoFisher Scientific, Waltham, MA, USA). The phase composition of sorbent prior and after lead sorption was checked by XRD (PANalytical Aeris with Co Kα X-ray tube, 40 kV, 75 mA, PANalytical, Almelo, the Netherland), the phase identification was done by help of PDF-2 database. The surface distribution of sorbed Pb was examined by Scanning Electron Microscope (SEM) Phenom XL (Phenom World, Eindhoven, the Netherland) with EDS (Energy-Dispersive X-ray spectroscopy) detector. The initial part of hydration process in cement pastes was monitored by isothermal calorimetry (homemade heat flow calorimeter KC01) [38]; two replicates were done. The compressive strength of pastes (after 3, 7, 14 and 28 days of curing) was determined by help of MTS Criterion 43 device with controlled speed of deformation (0.2 mm/s). Three samples were used for strength determination. In order to characterize composition changes of individual materials, the samples of pastes after the compressive strength measurement were examined by thermal analysis (Labsys EVO, rate of heating 5 °C/min, Ar atmosphere, SETARAM Instrumentation, Caluire, France). The initial part of hydration (3 and 7 days) was studied also by help of ^27^Al Magic Angle Spinning Nuclear Magnetic Resonance Spectroscopy (MAS NMR). Solid-state NMR (ssNMR) spectra were recorded at 11.7 T magnetic field using an AVANCE III HD spectrometer (Bruker, Karlsruhe, Germany), at Larmor frequencies ν(^27^Al) = 130.336 MHz using a 4 mm cross-polarization (CP) magic angle spinning (MAS) probe. MAS spinning speeds for all samples were 11 kHz. The ^27^Al MAS NMR isotropic chemical shift was calibrated using Al(NO_3_)_3_ in H_2_O as an external standard and referenced to 0 ppm. Single pulsed experiments were performed with a pulse length of 2.5 µs at 200 W at a recycle delay of 2 s. 512 scans were collected in each experiment. SPINAL64 decoupling sequence was applied for suppression of strong dipolar interactions. Dried samples were placed into ZrO_2_ rotors. All NMR experiments were performed at laboratory temperature [39]. In order to stop the hydration progress in samples between the strength determination and the real time of portlandite content (TG) and NMR analysis, the specimens were crushed in agate mortar with isopropanol which removed the free water; the samples were kept in isopropanol for 24 h, than filtered, mixed with diethyl-ether and finally filtered, dried at laboratory temperature and stored in sealed containers in desiccator.

## 3. Results

### 3.1. Speciation of Sorbed Lead

**Figure 1:**
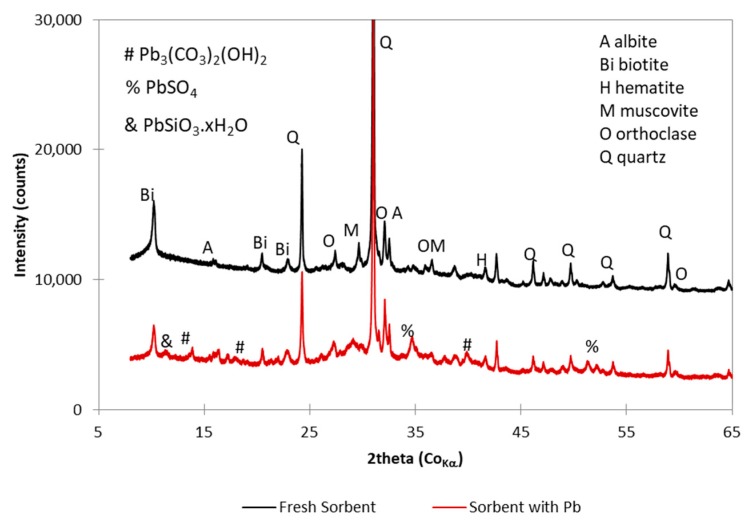
Diffractograms of fresh sorbent and sorbent with sorbed lead.

The unused ceramic sorbent is very complex material containing quartz, albite, biotite, orthoclase, hematite, as well as amorphous matter having its origin in thermal decomposition of illitic clay which was used as primary raw material for brick blocks production (Figure 1). The Pb sorption took place according to Langmuir isotherm [37], which assumes formation of monolayer of adsorbate on the surface. Nevertheless, three lead containing phases were identified by XRD in the spent sorbent: hydrocerussite Pb_3_(CO_3_)_2_(OH)_2_, anglesite PbSO_4_ and an ill-defined hydrated lead silicate PbSiO_3_·*x*H_2_O.

**Figure 2:**
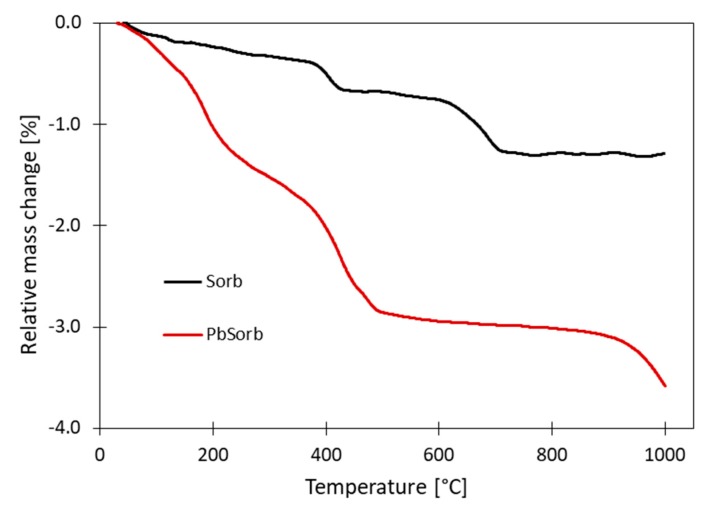
Thermogravimetry of fresh and used sorbent.

**Figure 3:**
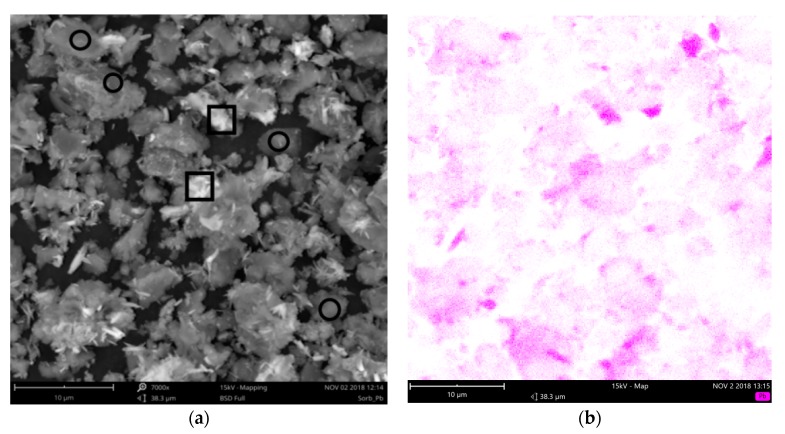
SEM micrograph (**a**) of ceramic sorbent with sorbed lead (PbSorb) and EDS analysis of Pb distribution (**b**) on the surface. Circles indicate areas with Pb content between 9 and 55% by mass; squares indicate areas with 60 and 80% Pb by mass.

In order to support the XRD results, the thermogravimetry and SEM experiments were carried out as well (Figure 2 and Figure 3). The unused sorbent reached mass loss only ca. 1.3% at 1000 °C. Since it is ceramic material, fired already in production at about 900 °C, its mass change to the temperature of firing theoretically should be zero. Nevertheless, there are two distinct steps at 400 °C and 650 °C. The XRD did not detect neither Ca(OH)_2_, CaCO_3_ nor any other calcium containing mineral in ceramic sorbent, while the XRF showed 15.4% of CaO. This means that this amount of CaO must be present in the amorphous portion of ceramic. The position of the mass change steps in TG corresponds to thermal decomposition of Ca(OH)_2_ and CaCO_3_; the observed mass changes indicate content of 1.2% of both Ca(OH)_2_ and CaCO_3_ in sorbent. The TG curve of PbSorb reached −3.6% of relative mass change. The dominant part corresponds to gradual thermal decomposition of Pb_3_(CO_3_)_2_(OH)_2_ [40]. The mass change around 650 °C is missing in PbSorb due to conversion calcium carbonate to hydrocerussite during the sorption experiment. The terminal part of TG curve indicates beginning of evaporation of volatile lead compounds.

The presence of lead on the surface of sorbent was also examined by SEM (Figure 3). The EDS map shows that Pb is present on the entire surface of the spent sorbent, but in varying concentration. The areas with high intensive magenta color contain ca. 80% of Pb and 3% of C (by mass), what corresponds well with the hydrocerussite composition; the BSD (backscattered electron imaging) image illustrate its crystals (in squares). The rest of surface is covered by less concentrated Pb (9–55% by mass) adsorbed on the ceramics. This letter form corresponds to a monolayer assumed by well-known Langmuir isotherm [17].

### 3.2. Calorimetry

**Figure 4:**
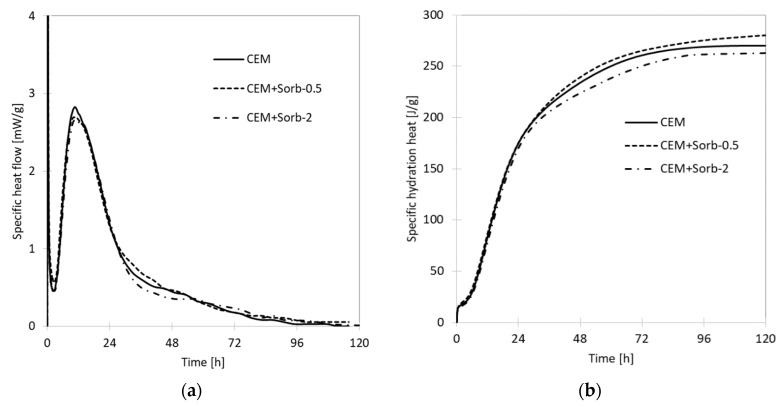
Isothermal calorimetry curves of OPC and its mixtures with ceramic sorbent (without Pb). (**a**) specific heat flow curves, (**b**) specific hydration heat.

Isothermal calorimetry provides a fundamental insight on the kinetics of the hydration process. Firstly, the influence of ceramic sorbent on hydration of OPC was tested (Figure 4); the specific heat flow and cumulative hydration heat curves proved that the used amount sorbent has not any significant effect on hydration course. The heat evolution is dominated by a main hydration peak which appeared 3 h after water adding. The induction period (time between initial and main hydration peaks) took ca. 2 h in all cases. The measureable hydration heat evolution terminated after four days and all mixtures provided similar values of hydration heat (263, 270, and 280 J/g).

**Figure 5:**
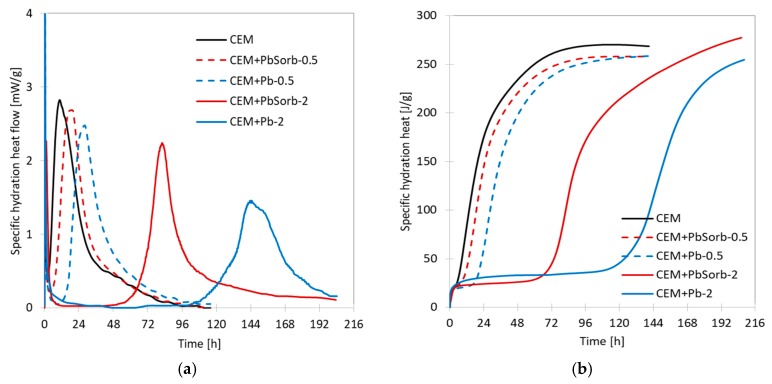
Isothermal calorimetry curves of Pb containing mixtures. (**a**) specific heat flow curves, (**b**) specific hydration heat.

The presence of lead influenced the hydration course (Figure 5) of cement pastes. The admixing of PbO prolonged extremely the induction period to ca. 14 and 100 h (for 0.5 and 2% respectively). Consequently, the hydration heat evolution took place more than 200 h in CEM + Pb-2 system. The total evolved hydration heat was practically unchanged when compared to OPC. The negative effect on hydration rate was suppressed to a half when the lead was dosed in sorbed form; in this case, the induction period of CEM + PbSorb-0.5 was 6 h and 44 h in the more concentrated system.

### 3.3. Time Evolution of Compressive Strength and Composition of Pastes

**Figure 6:**
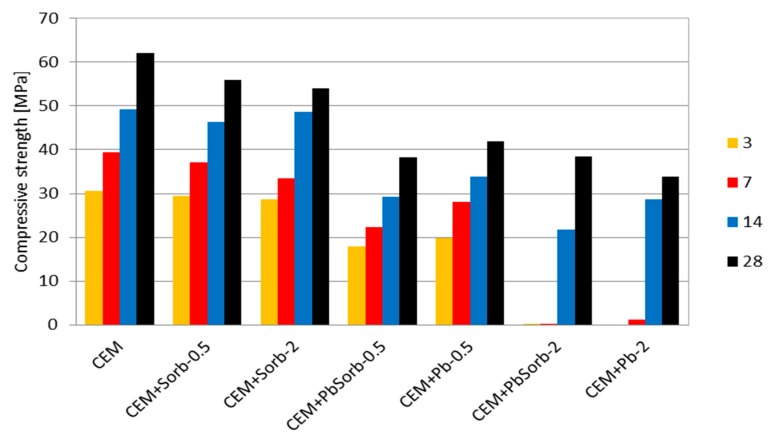
Compressive strength of pastes in time.

The time evolution of strength is of primary importance when a cementitious system is intended to be used as construction material or even as solidificate. The reference paste CEM reached a typical increase of strength in time, as would be expected for OPC (Figure 6). The addition of ceramic powder did not influence the strength evolution much, just a slight decrease of hardening rate can be observed due to presence of ceramic pozzolana in paste. The both mixtures with 0.5% of lead achieved comparable strength to each other, but when compared to CEM or CEM-Sorb, the strength was lower by ca. 30%. The strength of mixtures with 2% of Pb was obviously impacted negatively by their delayed hydration; the three-day strength was unmeasurable and seven-day strength was very low. In later ages, the strength was comparable with 0.5% mixtures and again, there was no clear difference between PbO or PbSorb.

**Figure 7:**
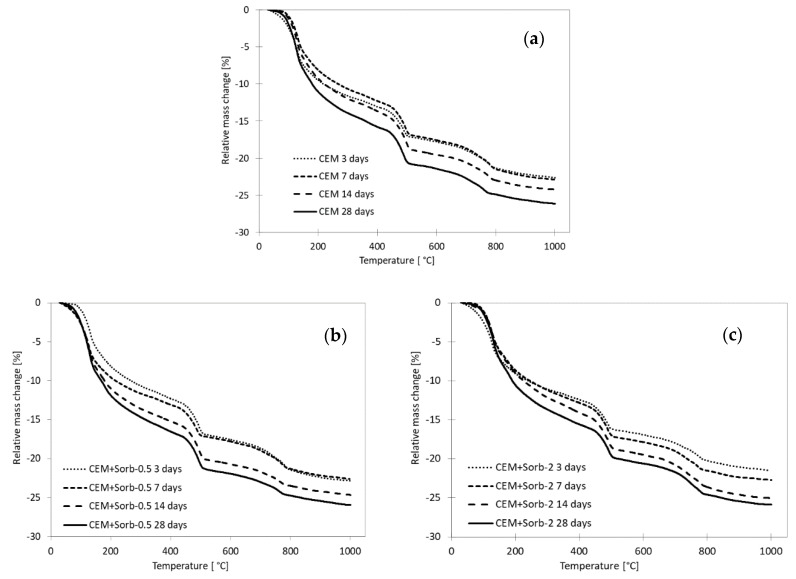

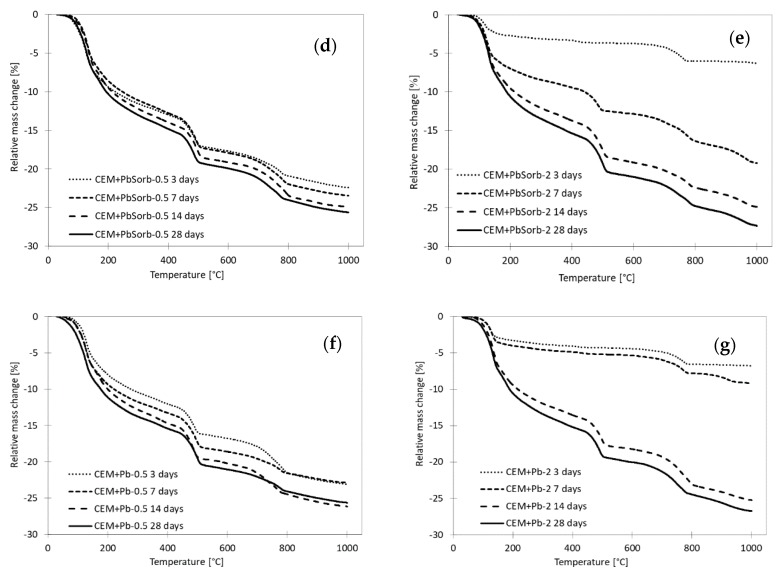
Thermogravimetry of pastes under study; (**a**) CEM; (**b**) CEM + Sorb-0.5; (**c**) CEM + Sorb-2; (**d**) CEM + PbSorb-0.5; (**e**) CEM + PbSorb-2; (**f**) CEM + Pb-0.5); (**g**) CEM + Pb-2.

**Figure 8:**
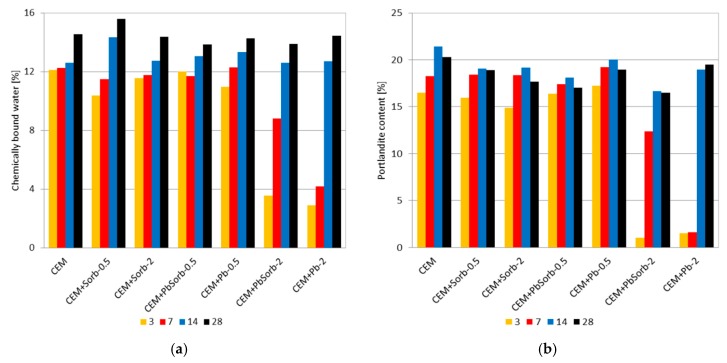
Time evolution of chemically bound water (**a**) and portlandite content (**b**) in pastes.

The amount of hydration products generated in a paste was quantified by thermogravimetry (Figure 7). This approach is able to quantify formation of hydration products in individual mixes and thus to compare the hydration delay in mixtures. The amounts of C-S-H and C-A-H hydrates were determined indirectly by help of “chemically bound water” (CBW) released during the heating from laboratory temperature up to beginning of portlandite (Ca(OH)_2_) decomposition (between 430 and 530 °C, the exact interval was determined for each sample individually by help of realtive mass change derivative). Since the free water was removed form the samples by help of isopropanol extraction, the CBW correspond simply to the relative mass change from beginning of experiment to start of portlandite decomposition. The portlandite amount in the sample was determined on basis of relative mass change in the above specified interval, divided by factor 0.2434 (i.e., ratio of molar weight of water and portlandite) (Figure 8). The portlandite formation was suppresed in both mixtures with 2% Pb in three and seven days, but the PbSorb had less delaying effect than PbO. The amount of CBW in control mixture CEM reached 12% after three days of hydration and did not change much until 14 days; just 28 days caused its increase to 15%. The mixtures with unsused ceramic sorbent were characterized by more gradual increase of CBW, as well as mixtures with 0.5% Pb. The different behavior was observed in mixtures with 2% Pb; even though strength and calorimetry indiated that any change took place in these systems in three days, there was certain amount of CBW and portlandite produced already. At 14 and 28 days, the CBW and portlandite content was comparable with the other pastes. Certain decrease of portlandite content in 28 days in most of the mixtures was caused by portlandite consumption in carbonation and pozzolanic reaction with the ceramic sorbent.

**Figure 9:**
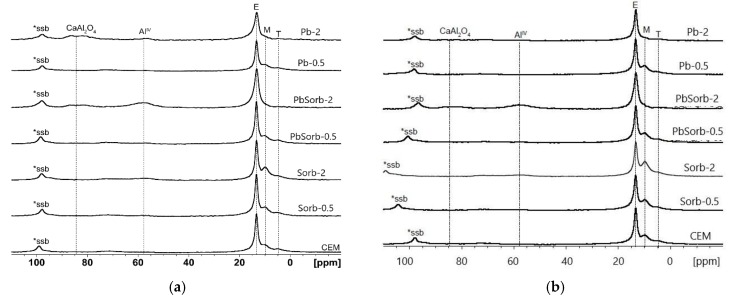
^27^Al MAS NMR spectra recorded in studied pastes after three (**a**) and seven (**b**) days of hydration. The spinning sideband signals are marked as *ssb. CaAl_2_O_4_ is shown as example of aluminates present in OPC. E is ettringite (Ca_6_[Al(OH)_6_]_2_(SO_4_)_3_·26H_2_O); M monosulfate (Ca_4_[Al(OH)_6_]_2_SO_4_·6H_2_O) and T “third aluminate phase”.

The solid state ^27^Al MAS NMR was used in order to evaluate the hydration process of aluminates present in OPC and in ceramic sorbent (Figure 9). The ^27^Al chemical shift of Al^IV^ species in anhydrous calcium aluminates, present in OPC, ranges from 69 to 86 ppm [41]. The ceramic sorbent contains also Al^IV^ sites (δ_iso_ = 58 ppm) which have its origin in dehydroxylated illite [42]. Both of these types of Al^IV^ sites were fully hydrated already after three days (Figure 8) in pastes, except those with 2% of Pb. The aluminous hydration products contain Al^VI^ sites with chemical shift between 5 and 15 ppm [43]. Three distinct signals were obtained; the most intensive (E) belongs to ettringite (Ca_6_[Al(OH)_6_]_2_(SO_4_)_3_·26H_2_O), the other to monosulfate (Ca_4_[Al(OH)_6_]_2_SO_4_·6H_2_O) (M) and “third aluminate phase”, described in [43] as “less-crystalline aluminate gel or calcium aluminate hydrate”. The spectra of Pb-2 and PbSorb-2 of both ages contain just ettringite; the other aluminate phases are absent. In accordance with published data on Al^IV^ hydration—the signal E appeared after a few hours of hydration, while the M and T signals grown later [43]. The main difference between three and seven days of spectra lies in vanishing of signal at 85 ppm in all samples; a certain amount of unreacted “ceramic” Al^IV^ species remained in PbSorb-2.

### 3.4. Leaching of Pb from Sorbent and Cement Pastes

The leaching of lead from the used sorbent PbSorb (Table 3) reached rather high value; hence the sorbent must be stabilized in order to prevent lead leaching. The Pb containing cement pastes, studied within this paper, were subjected to leaching tests. In all cases, the immobilization was successful; the leaching of Pb was reduced by four orders of magnitude. The measured values of leaching (in mg/kg of paste) proved that Pb stabilization took place; i.e., the leaching reduction is not only due to dilution of Pb content after mixing with OPC. According to European standard [44], wastes are classified to three classes: I—inert, II—other, and III—hazardous; the classification parameter is leachate concentration—in case of lead 0.05 and 1 mg/L. It means that sorbent with lead is “hazardous waste”, pastes with 0.5% Pb are “other waste” and the pastes with 2% belong to ‘hazardous waste’ as well.

## 4. Discussion

The waste ceramic powder, generated in brick blocks grinding to desired dimensions, proved high sorption capacity for Pb (121 mg/g); this value is comparable or higher than those of common low cost sorbents, such are clay minerals or zeolites [12,13,14]. The sorption of Pb on the sorbent surface took place via two processes; firstly, the entire surface of the sorbent was covered by adsorbed Pb layer as was shown by SEM-EDS, which corresponds to monolayer assumed by Langmuir isotherm. Secondly, hydrocerussite (basic lead carbonate Pb_3_(CO_3_)_2_(OH)_2_) crystals precipitated locally on sorbent particles. Freundlich isotherm is assuming an “infinite” formation of sorbate layer on the surface; the observed bimodal Pb sorption behavior can be understood as case between these two extremes. The amount of precipitated hydrocerussite is limited by carbonate content in sorbent, thus the total sorption capacity is limited as well and the data obey Langmuir isotherm, even though there is more Pb sorbed than would be in a monolayer. The formation of hydrocerussite was enabled by basic character of ceramic sorbent—it contains, in its amorphous portion, certain amount of calcium hydroxide and calcium carbonate, as revealed thermal analysis of ceramics, as well as its certain ability to hydrate without any external activation [45]. The intensive leaching of Pb from the sorbent caused its classification as “hazardous waste”, thus the immobilization of sorbent became a necessity. The ceramic powder, thanks to its chemical composition, is pozzolanic material able to replace part of the OPC in concrete [23,42]. Calorimetry, as well as the compressive strength measurement, proved that ceramic sorbent by its own did not change the hydration course or the achieved strength.

The calorimetry results on PbO + OPC mixtures are in agreement with those published on this system [29]; the increasing content of PbO caused lengthening of induction period. When the lead was added in sorbed form, the induction period lengthening was reduced to ca. 50% compared to PbO. The less pronounced effect of sorbed lead compared to PbO is related to solubility of lead compounds in basic environment of hydrating cement; PbO forms soluble plumbate, which hinders hydration of C_3_S in cement [25], while hydrocerussite is stable in basic environment [46] and does not interact with hydration process. Unfortunately, hydrocerussite involves just part of the sorbed lead and thus PbSorb influence on hydration is weaker compared to PbO, but not fully neutral. Thermogravimetry and MAS NMR complemented the calorimetry measurement to the total hydration time 28 days. The lower Pb dose (0.5%), regardless the lead form (PbO or PbSorb), did not influence the CBW, portlandite content (TG) nor hydrated aluminates (NMR), but reduced the strength (Figure 5); it means that even though the above mentioned chemical characteristics were not changed, the binding ability of C-S-H gel was reduced. It is in agreement with finding in [32], that Pb is incorporated to the hydration products. At the higher Pb dosage (2%), the form of Pb addition matter in the same way as in calorimetry. TG results on 2% systems clearly evidenced the delay of hydration products formation. The sorbed lead caused less retardation of portlandite and CWB growth compared to PbO. At the longer times (14 and 28 days), the portlandite and CBW content were identical to the other systems, while the strength growth was delayed more than in 0.5% systems. Again, it indicates a changed structure of hydration products due to Pb incorporation.

The immobilization of ceramic sorbent was successful, but the 2% systems still fall within hazardous waste, even though the lead concentration in leachate was obviously much lower than in the case of non-stabilized sorbent. The obtained results, dealing with both the hydration process and lead immobilization, revealed that 2% of Pb per OPC is a dose that significantly influences the paste behavior in both hydration and leaching aspects.

## 5. Conclusions

The influence of lead sorbed on ceramic sorbent on the hydration process of Ordinary Portland Cement was studied by help of calorimetry, thermogravimetry, and MAS NMR spectroscopy. The findings can be briefly summarized:

▪ The ceramic sorbent reached high sorption capacity for lead (121 mg/g). Lead was partially adsorbed on the surface and partially precipitated as basic lead carbonate (hydrocerussite, Pb_3_(CO_3_)_2_(OH)_2_).
▪ The hydration retardation caused by sorbed lead was less pronounced than the action of PbO. The reason lies in formation of hydrocerussite, which is more stable in basic environment than PbO, which forms plumbate ions.
▪ The 0.5% Pb by mass, regardless its form, is reducing the strength of cementitious paste moderately, while the 2% dosage is extremely retarding the hydration and strength evolution.
▪ The leaching experiments also showed, that 0.5% Pb can meet the “other waste” requirement, while 2% of pastes fall already to “hazardous waste”.
▪ The results indicate that Pb is absorbed to structure of hydration products. The structure of C-S-H with Pb incorporated is a challenge for future research.

## Figures and Tables

**Table 1:** Composition of studied pastes (in g per 100 g of Ordinary Portland Cement).

Paste	OPC	Sorb	PbSorb	PbO	Water
CEM	100	-	-	-	48
CEM + Sorb-0.5	100	3.6058	-	-	48
CEM + Sorb-2	100	14.4231	-	-	48
CEM + PbSorb-0.5	100	-	4.1446	-	48
CEM + Pb-0.5	100	-	-	0.5388	48
CEM + PbSorb-2	100	-	16.5782	-	48
CEM + Pb-2	100	-	-	2.1552	48

**Table 2:** Composition (% by mass) of raw materials.

Component	CEM I 42.5 R	Sorb	PbSorb
SiO_2_	18.7	49.9	46.9
Al_2_O_3_	4.2	20.4	18.8
Fe_2_O_3_	3.4	5.0	4.8
CaO	65.9	15.4	9.5
MgO	1.3	2.8	1.7
K_2_O	0.8	3.3	3.1
Na_2_O	0.2	0.5	0.4
TiO_2_	0.3	0.8	0.8
SO_3_	4.3	1.5	1.0
PbO	0.05	0.04	13.0
Cl	0.06	0.00	0.00

**Table 3:** Leaching of Pb from sorbent and cement pastes.

Paste	Pb Content	Leachate Conc.	Leaching	
	mg/kg	mg/L	mg/kg	%
PbSorb	120,680	1370	13,700	11.35
CEM + Pb-0.5	4975	0.33	3.3	0.07
CEM + Pb-2	19,585	1.81	18.1	0.09
CEM + PbSorb-0.5	4803	0.35	3.5	0.07
CEM + PbSorb-2	17,162	1.36	13.6	0.08
